# Urban Bird Feeding: Connecting People with Nature

**DOI:** 10.1371/journal.pone.0158717

**Published:** 2016-07-18

**Authors:** Daniel T. C. Cox, Kevin J. Gaston

**Affiliations:** Environment and Sustainability Institute, University of Exeter, Penryn, Cornwall, TR10 9EZ, United Kingdom; Cornell University, UNITED STATES

## Abstract

At a time of unprecedented biodiversity loss, researchers are increasingly recognizing the broad range of benefits provided to humankind by nature. However, as people live more urbanized lifestyles there is a progressive disengagement with the natural world that diminishes these benefits and discourages positive environmental behaviour. The provision of food for garden birds is an increasing global phenomenon, and provides a readily accessible way for people to counter this trend. Yet despite its popularity, quite why people feed birds remains poorly understood. We explore three loosely defined motivations behind bird feeding: that it provides psychological benefits, is due to a concern about bird welfare, and/or is due to a more general orientation towards nature. We quantitatively surveyed households from urban towns in southern England to explore attitudes and actions towards garden bird feeding. Each household scored three Likert statements relating to each of the three motivations. We found that people who fed birds regularly felt more relaxed and connected to nature when they watched garden birds, and perceived that bird feeding is beneficial for bird welfare while investing time in minimising associated risks. Finally, feeding birds may be an expression of a wider orientation towards nature. Overall, we found that the feelings of being relaxed and connected to nature were the strongest drivers. As urban expansion continues both to threaten species conservation and to change peoples’ relationship with the natural world, feeding birds may provide an important tool for engaging people with nature to the benefit of both people and conservation.

## Introduction

Globally, biodiversity and natural habitat continue on trends of apparently inexorable loss [1]. This is at a time when researchers are increasingly recognizing the broad range of physical, mental and social benefits that interacting with nature provides to people (e.g. [2–4]). As both a greater number and proportion of us live in cities there is growing concern that many people are becoming disengaged from the natural world (termed the ‘extinction of experience’; [5–6]). This is potentially serious, because it may lead, first, to a loss of people’s desire to interact with nature, so cutting them off from the associated benefits [6–8], and second, to a reduction in broad-based public support for biodiversity conservation [6,9–11], because people’s awareness of environmental issues is influenced crucially by their experiences of nature in everyday surroundings [12]. However, despite the oft-reduced opportunities, many people still seek out regular interactions with nature (e.g. [13,14]). Strengthening understanding of the motivations behind why they do so may be key both to maximising the benefits, and harnessing support for broader conservation issues.

For many people, particularly those living in urban areas, their interactions with wild birds may form the main wildlife interactions that they experience in daily life [15]. So, it is perhaps unsurprising that despite the widespread extinction of experience there is frequent provision of food by people for garden birds. This is often the most common form of wildlife gardening, with around a half of urban households in some western countries putting out food on a regular basis (estimated from [16–21]). The large scale provision of supplementary food for wild birds has significant ecological (reviewed by [22]) and economic [23] impacts. Increasingly it is also being recognised as being an important potential tool for stimulating a broader interest in the natural world [7,17,23–26]. However, despite the clear importance that feeding wild birds has for both birds and people there is still no clear understanding of people’s motivations for doing so. Here we distinguish three possible mechanisms, namely potential psychological benefits from watching wild birds; a concern about the welfare of wild birds; and/or as a more general orientation towards interacting with nature.

The psychological benefits that people receive from watching birds in their garden, such as feelings of pleasure, are the most obvious motivation for feeding them [21–27]. We explore two such benefits that might drive garden bird feeding. First, attention restoration theory proposes that the natural world promotes recovery from mental fatigue and offers opportunities for reflection [28], while stress reduction theory indicates that natural environments facilitate reductions in physiological arousal following stress [29]. Both of these theoretical frameworks promote relaxation thus leading to reduced stress and improved mental health (e.g. [30–31]). Watching birds and their behaviour as a visible component of nature may contribute significantly to these feelings. Second, watching garden birds may provide people with a feeling of being connected to nature, contributing towards an increased sense of belonging in the natural world (reviewed [32]). How a person relates to nature (i.e. how connected they are) has been shown to be a strong predictor of environmental attitudes (reviewed in [32]), and has been positively associated with subjective well-being [33–34] and reduced anxiety [35].

Traditionally, and currently, people in the northern hemisphere more often provide food for birds in winter when they are perceived to need more assistance with resources [23,27,36]. This is despite daylight hours being shorter, with people spending less time in their gardens and so arguably there being less likelihood of viewing the birds directly. This suggests that a concern about bird welfare may be an important motivation behind providing food. Indeed, many people feel passionately about their birds and are keen to learn best feeding practises. The aggregation of large numbers of birds around a food source has been associated with an increased risk of disease transmission [22], and best practice guidelines recommend that this risk can be reduced by the regular cleaning of feeders (e.g. [37]). However, this entails a time investment and because householders often cannot see the effects of disease transmission it may have little visible effect. Therefore people who clean feeders can be regarded as showing an increased concern for bird welfare.

Finally, there is increasing evidence that some people are more orientated towards interacting with the natural world than others [4,6,8], and are willing to invest more to obtain this interaction even when they have a reduced opportunity for doing so [38,39]. It can be relatively easy to attract birds to a feeder through the provision of food. We explore whether the mechanism behind people doing so is either a response to the opportunity of birds already present in the garden, or some people being orientated towards specifically attracting birds even when there are none. Indeed, a bird feeder plays a unique role in attracting birds to a focal location where they can be viewed more closely and for longer periods. People who invest in maintaining a bird feeder, so seeking the closer interaction provided, might be seen to be more orientated towards interacting with nature through bird feeding.

Here we ask survey respondents to rate three Likert statements as components of each motivation, to explore the degree to which they drive people’s bird feeding activities: the psychological benefits they receive; their concern about bird welfare; and/or as a way to express their general orientation towards interacting with nature.

## Materials and Methods

### Ethical statement

This research was conducted with approval from, and in accordance with, the University of Exeter Biosciences ethical review committee, project number 2013/320. Before completing the survey respondents were asked to provide written consent by checking a box stating their agreement to participate in the survey. Respondents were also asked to confirm that they were over 18 years of age. On the written consent form, participants were told that data would remain anonymous and would be protected and stored in a secured format. There is an electronic log of consent procedure to document the process.

### Survey methods

We surveyed garden bird feeding activities and perceptions of common garden bird species using a questionnaire approach across three English towns located, in close proximity (~60 km to the north of London, UK): Milton Keynes (52°02’N, 0°45’W), Luton (51°53’N, 0°25’W) and Bedford (N52°58’N, 0°28’W). These each have sizeable human populations of, respectively, c. 230,000, c. 240,000, and c. 160,000 (2011 Census, UK). Two general survey methods were used. First, between November 2013 and February 2014, 20 households were selected at random in each of the three towns. A researcher knocked on the doors of the houses and asked one member of the household to complete the questionnaire. The survey participant in each household was also asked to enlist two other known households from within ~500m to participate in the survey. Potential participants were contacted by email or phone and the questionnaire was delivered by hand. Second, between March and July 2014 up to ten streets in each town were selected at random. A researcher then knocked on the doors of all houses with evidence that someone was home, e.g. from a car in the drive. The project was explained to the resident, who was then asked to complete a questionnaire in his or her own time. In order to minimize possible bias resulting from certain groups being more likely to be at home, different streets were targeted at different times of day either late morning (11:00 to 13:00), mid afternoon (14:30 to 16:00) or late afternoon (17:00 to 18:30). Surveys were conducted at both weekdays and at weekends. For both survey methods a first attempt to collect the questionnaire was made two days after delivery, and if unsuccessful a subsequent attempt was made two days after that. One hundred and forty responses were collected by the first survey method, and 191 by the second. The survey was conducted in a stratified random way because we were not interested in the proportion of the urban population who fed birds, but instead wanted to understand the reasons those that fed birds had for doing so, whilst having a sufficiently large sample of people who did not feed birds for comparison purposes.

### Questionnaire design

We developed a questionnaire to explore people’s knowledge and experience of, and attitudes towards, garden bird feeding. The questionnaire took approximately six minutes to complete and consisted of close-ended questions. Only those questions used in the analyses reported here are discussed (See Tables A-E in S1 File for a fuller description of the questionnaire). To explore respondents’ motivations behind garden bird feeding, we asked people to rate the extent to which they agreed with each of nine statements. Responses were given on a five-point Likert scale, from strongly disagree to strongly agree. Three statements related to the psychological benefits that people obtain from watching birds in their garden (Table B in S1 File). These stemmed from known psychological benefits of interacting with nature (e.g. [28,40]). A further three statements explored perceived welfare benefits and a respondents’ willingness to minimise potential risks associated with bird feeding (Table C and D in S1 File). Finally, three statements assessed respondents’ orientation towards bird feeding over their opportunity for doing so, and the role that a bird feeder plays in this (Table D in S1 File). Five of the above statements related to bird feeding generally and were completed by all respondents, while four related directly to bird feeding activities and so were not completed by people who did not feed birds. Item phrasing can influence outcomes, and statements were designed to be neither strongly positive nor negative, nor to lead respondents. We also collected data on the socio-demographic status of the respondents, along with information on their bird feeding activities and their general awareness of the birds around them (Table A in S1 File). To try and understand why some people don’t feed birds, we also asked people who did not do so to score the Likert statement ‘I am not interested in feeding birds’, and why those that engaged in some form of bird feeding activity don’t do so more regularly ‘I don’t always remember to put out food’. As a crude measure of the independence of surveying multiple people from each street we also asked people to score the five point Likert statement ‘I feed birds because my neighbours do’. See Table C and D in S1 File.

Prior to statistical analysis we created a three-level factor pertaining to how regularly a household provided food for birds: regularly (those that replied daily or weekly), irregularly (those that replied monthly or less than once a month) or never (those that didn’t feed birds). Second, as a measure of people’s awareness of the birds around where they live and work, respondents were also asked to select one or more periods during the day when they usually noticed birds (the day was divided into four approximately equal periods; morning, lunchtime, afternoon and evening). We then constructed a second factor on a scale of 0–4 according to what proportion of their average day people reported noticing birds (e.g., someone who reported that they notice birds in the morning and afternoon would be given a score of 2). Those that answered ‘I don’t notice birds’ were given a score of zero. We created a third factor on gender (male/female). Respondents were asked their age within a five-year window, we then developed a fourth factor with ages pooled from 20 to 40 years, 40 to 60 years and >60 years. Finally we controlled for gross annual income by obtaining the ‘expected’ income categories for each postcode in which respondents resided (Office for National Statistics, Small Area Income Estimates 2007/08, Gov UK). These were then included as a four-level factor.

### Statistical analyses

All analysis was conducted in R 3.1.2 [41]. We did not find a difference in responses between the two methods of data collection (coefficient = 0.02 ± 0.04 (SE), p = 0.7), so we pooled responses from each (Appendix A in S1 File) and from the three towns. For any completed questionnaire, if any of the questions were incomplete, then that respondent’s question was removed from the analysis. Generalized Variance Inflation Factors (GVIFs) were used to check for multi-collinearity between factors, and found to be within acceptable norms, with all GVIFs <1.3. To determine whether bird feeding activities, bird awareness, age, gender and/or income were important predictors of answers to each of the nine statements we used ordinal regression models using the ‘ordinal’ package [42]. We then applied an Information Theoretic approach that simultaneously evaluates hypotheses by balancing between model complexity and goodness of fit [43]. We used the ‘MuMIn’ package [44] to produce all subsets of models based on the global model and rank them based on AICc. Following [45], and to be 95% sure that the most parsimonious models were maintained within the best supported model set, we retained all models where **Δ**AIC_c_ < 6. We then used model-averaging to produce the average parameter estimates of each parameter [43]. We used the ‘HH’ package to produce the Likert plots [46].

Based on the statements behind each motivation, we estimated which motivation was the strongest driver of bird feeding (i.e. which motivation had the strongest support). For each statement a score of 1 corresponded to strongly disagree, a score of five to strongly agree. Where necessary we then reversed the scores of statements so a high score always indicated support for bird feeding and/or welfare. Answers from all nine statements were then pooled, before building a mixed effects ordinal regression of the statement score (five level factor of one to five) against whether the statement represented a psychological benefit, welfare issue or orientation towards feeding birds (three-level factor). We controlled for the actual level of bird feeding activities because people who feed birds are likely to have stronger motivations for doing so. We included a unique ID for each respondent as a random effect.

## Results

### Respondents

**Fig 1:**
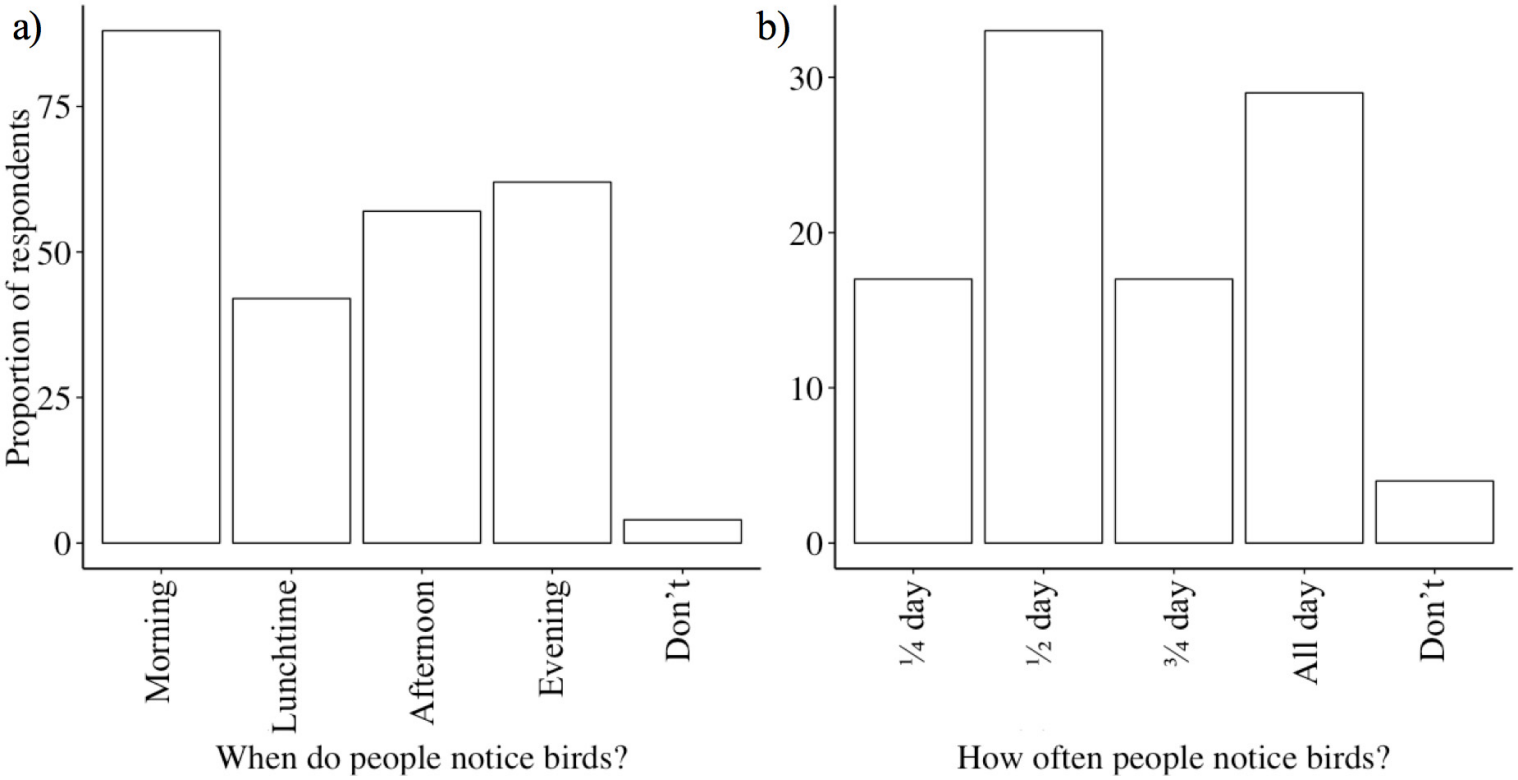
Summary statistics from 331 respondents, showing the proportion of: a) respondents that noticed birds during different periods of the day and, b) the proportion of the day that most people noticed birds.

A total of 331 questionnaires were completed and used in the analysis (140 and 191 completed from each survey method, respectively). For the first survey method we received a response rate of 94%. For the second survey method, 90% agreed to participate in the survey, of these 87% completed the survey giving an overall return rate of 78%. We found that 89% of respondents answered strongly disagree or disagree to the statement ‘I feed birds because my neighbours do’ (average score 1.4 ± 0.8 (SE)). Although this is not conclusive it does indicate that people believed that they were acting independently and so we deemed that surveying multiple households from the same street did not confound the study. There was an over representation of female respondents (56% compared to 51% in Buckinghamshire and Bedfordshire county’s, 2011 Census) and of respondents over 60 years (42% compared to 28% in Buckinghamshire and Bedfordshire county’s, 2011 Census; Table Fa in S1 File). We found that 83% of households put out bird food, with 72% of those feeding birds doing so regularly (Table Fb in S1 File). The proportion of respondents who put out food did not vary by season (*χ*^*2*^ = 4.2, df = 3, p = 0.2). People most commonly noticed birds in the morning and evening (*χ*^*2*^ = 5.7, df = 3, p <0.0001; Fig 1a), while respondents tended to notice birds for different proportions of the day (*χ*^*2*^ = 86.9, df = 4, p <0.0001; Fig 1b) with only 29% of respondents noticing birds at all times of day (Fig 1b; acknowledging that individual respondents could score more than one period of the day). A logistic regression of feeding regularity against age, showed that people were more likely to feed birds regularly as they got older (estimate = 1.7 ± 0.4 (SE), p <0.0001).

### Motivations behind bird feeding activities

**Fig 2:**
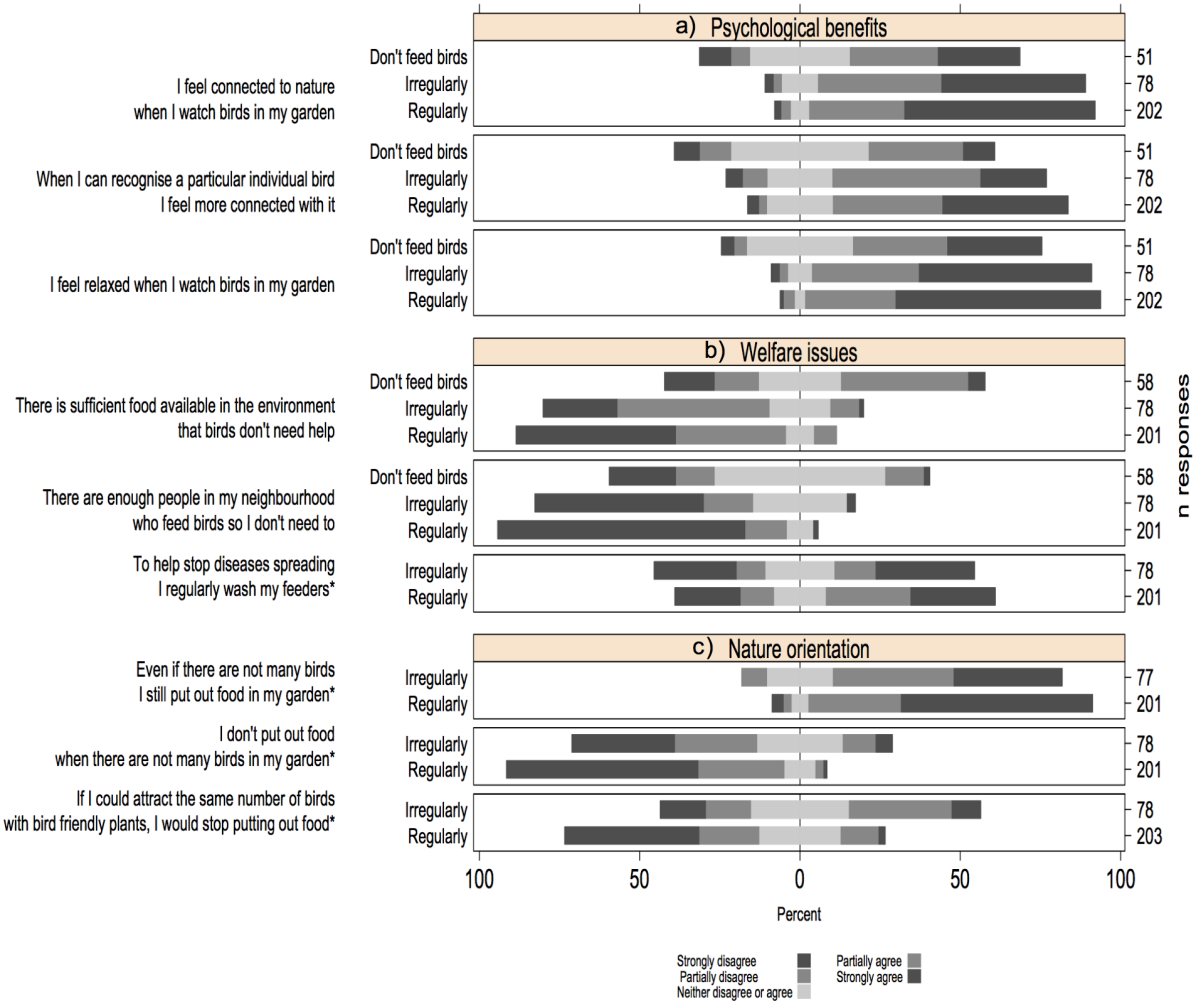
Three Likert statements as components of each of three different motivations behind garden bird feeding; a) psychological well-being benefits, b) a concern about avian welfare and c) nature orientation. For each statement we plotted the respondent’s score (strongly disagree to strongly agree) against how regularly they fed birds, because across statements this was the most consist predictor of motivation (* Statements 6–9 were only completed by people who fed birds).

Testing for assessed psychological benefits, we found that most people felt relaxed and connected to nature when they watched birds in their garden (Table 1a–1c, Fig 2). The feeling of being relaxed and connected to nature increased with the level of bird feeding activities (Fig 2a), and in people who noticed birds for a greater proportion of the day (Table 1a–1c). The feeling of relaxation also increased in respondents over 40 years old (Table 1a–1c).

**Table 1:** Ordinal regression of responses to three Likert statements as components of each of three motivations behind why people feed birds, a) psychological benefits, b) welfare issues, or c) nature orientation. We show model-averaged coefficients and standard errors in brackets. Given the ordinal nature of the predictor variables the results show the outcome as compared to a base factor level (shown in second row of table).

Statement	Parameter estimates (standard errors) for factor levels relative to a base factor level (shown below)											
	Don’t feed birds		Don’t notice birds				Age 20–40 years		Female	Income <£590		
	Feed Irregul.	Feed Regularl.	Notice 1/4 of day	Notice 1/2 of day	Notice 3/4 of day	Notice all day	Age 40 to 60 years	Age > 60 years	Male	Income £591-£670	Income £671-£790	Income >£791
*a) I feel connected to nature when I watch birds in my garden*	1.0 (±0.4)**	1.2 (±0.3)***	0.6 (0.5)	0.8 (0.5)	1.3 (0.6)*	2.1 (±0.6)***	0.5 (0.3)	0.4 (0.3)	0.0005 (0.3)	0.3 (0.2)	0.3 (0.4)	-0.3 (0.9)
*b) When I can recognise a particular individual I feel more connected to it*	0.7 (0.4)*	1.3 (0.4)***	0.2 (0.5)	0.7 (0.5)	0.7 (0.5)	1.1 (0.5)	-0.2 (0.3)	-0.1 (0.3)	0.2 (0.2)	0.1 (0.2)	0.5 (0.3)	-0.4 (0.7)
*c) I feel relaxed when I watch birds in my garden*	1.1 (0.4)**	1.3 (0.4)***	1.3 (0.5)*	1.6 (0.5)**	1.6 (0.6)**	1.6 (0.5)**	1.0 (0.3)**	0.8 (0.3)*	0.2 (0.2)	0.008 (0.2)	0.9 (0.4)	-1.1 (0.9)
*d) There is sufficient food available in the environment so that birds don’t need help*	-1.4 (0.3)***	-2.4 (0.3)***	-0.8 (0.5)	-0.3 (0.5)	-0.8 (0.5)	-0.7 (0.5)	0.4 (0.3)	0.02 (0.3)	-0.3 (0.2)	0.3 (0.2)	0.4 (0.3)	0.2 (0.9)
*e) There are enough people in my neighborhood who feed birds*, *so I don’t have to*	-1.5 (0.4)***	-2.6 (0.3)***	#	#	#	#	0.04 (0.3)	-0.7 (0.3)*	-0.4 (0.3)	0.4 (0.3)	0.2 (0.4)	0.5 (0.8)
*f) To help stop diseases spreading I regularly wash my feeders*^+^	-	1.3 (0.3)***	-1.8 (1.0)	-2.4 (1.0)	-1.9 (1.0)	-2.3 (1.0)	0.8 (0.4)*	1.2 (0.4**)	-0.5 (0.2)	0.5 (0.3)	0.3 (0.3)	-2.1 (0.8)
*g) Even if there are not many birds I still put out food*^+^	-	1.0 (0.3)***	1.4 (0.8)	0.9 (0.7)	1.0 (0.8)	1.6 (0.8)*	0.3 (0.4)	0.7 (0.4)	0.07 (0.3)	-0.6 (0.3)	-0.2 (0.4)	-1.3 (0.9)
*h) I don’t put out food when there are not many birds in the garden*^+^	-	-1.2 (0.3)***	0.2 (0.9)	0.5 (0.9)	0.1 (0.9)	-0.5 (0.9)	0.6 (0.4)	-0.2 (0.4)	0.1 (0.3)	#	#	#
*i) If I could attract the same number of birds with bird friendly plants*, *I would stop putting out food*^+^	-	-1.4 (0.3)	-1.6 (0.8)*	-0.8 (0.7)	-1.7 (0.8)*	-1.7 (0.8)*	0.6 (0.3)	-0.3 (0.3)	0.1 (0.3)	-0.1 (0.3)	0.6 (0.4)	0.8 (0.8)

The significance of factor levels are shown as:

*P <0.05;

**P <0.01;

***P <0.001.

^#^ variable was not retained in the top models

^+^Statements were only scored by those people who fed birds (n = 280), thus irregular bird feeding became the base factor level.

Testing for welfare concerns, we found that the perception that there is not enough natural food available for birds increased with the levels of bird feeding and in respondents >60 years of age (Table 1d, Fig 2b). The sentiment that there are enough people in my neighbourhood who feed birds decreased with the frequency of bird feeding (Table 1d and 1e, Fig 2b). Overall, people who fed birds regularly and people over 40 years were more likely to invest time taking preventative measures against disease transmission by washing their feeders regularly (Table 1f, Fig 2b).

Finally, testing for orientation towards interacting with nature, we found that most people, but in particular those who put out food regularly, did so to try and attract birds to their garden, putting out food whether birds were present or not (Table 1g and 1h, Fig 2c). People who fed birds regularly were less likely to stop putting out food if they could attract the same number of birds with bird-friendly plants (Table 1i, Fig 2c). We did not find gender or income to be a significant predictor of any statement.

**Fig 3:**
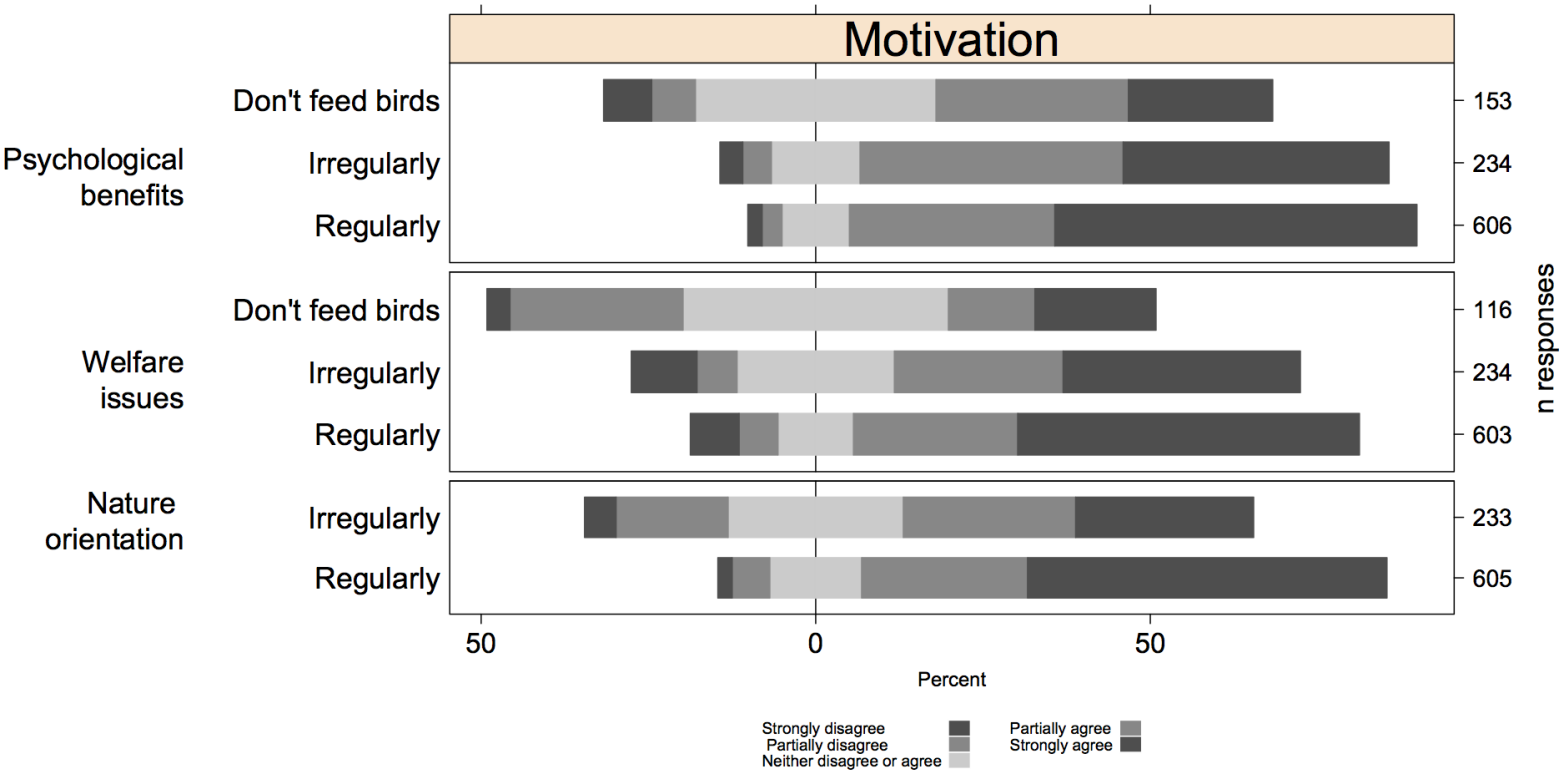
Likert plots for each of the three motivations behind garden bird feeding. Where necessary we reversed statement scores, so that a high score always indicates support for bird feeding and/or welfare. We then pooled statements by motivation.

A mixed effects ordinal regression of adjusted statement score against motivation, suggested that based on the statements, the psychological benefits were the strongest driver of bird feeding (Table 2, Fig 3). Nature orientation and a concern about avian welfare were equally strong motivations (Table 2, Fig 3).

**Table 2:** A mixed effects ordinal regression of statement score against motivation, while controlling for feeding activities. We included the respondent’s unique ID as a random effect. Coefficients show difference in motivation score relative to welfare, and bird feeding against those people who don’t feed birds.

*Factor level*	*Coefficient (CI)*	*t-value*
Psychological	0.35 (±0.08)	4.4***
Orientation	0.06 (±0.09)	0.5
Irregular feeding	0.81 (±0.19)	4.2***
Regular feeding	1.86 (±0.17)	10.8***

Significant factor levels are shown as:

***P <0.001.

Of the 56 people who never put out food for birds, 78% either disagreed or strongly disagreed with the statement ‘I am not interested in feeding birds’ (average score = 1.9 ±1.2 (SE)). People who fed birds regularly (estimate = -1.4 ± 0.3 (SE), p <0.0001) or were over 60 (estimate = -0.8 ± 0.4 (SE), p = 0.02) were less likely to forget to put out food.

## Discussion

In an increasingly urbanized world the on-going separation of people from nature, ‘the extinction of experience’, is considered by many both a major public heath risk [7,3] and a fundamental obstacle to halting and reversing the global biodiversity crisis [6,8]. It is a consequence of a behavioural shift towards people spending a greater proportion of time indoors or engaged in non-nature based activities [47,48]. This is also a period when the simple act of providing food for garden birds is increasing in popularity (e.g. [27]). Garden bird feeding has wider implications than supporting populations of often-common species, instead it is increasingly being recognised as an important component of many people’s daily nature interactions [7,15,23–26]. If so, bird feeders may make excellent ‘ambassadors’ for engaging people with nature and halting the extinction of experience. A small number of qualitative studies have started to explore the possible motivations behind the rapid increase in bird feeding [21,25–26,49], citing reasons such as feelings of pleasure [21,27] or deriving well-being by adopting a warden-like role to their wildlife [26]. However, despite the undoubted financial implications (see [27]) and impacts on avian welfare [22], it is still unclear why so many invest their time and money feeding birds. Here, we found that there were a variety of strong motivations, with evidence that the associated self-reported psychological benefits were the strongest driver (acknowledging that it is not possible to draw broader conclusions about these motivations beyond those from the individual statements; although we have mitigated much of the inherent bias within self-reported behaviour through large sample sizes and an ordinal regression analytical approach, a degree of caution must be exercised when interpreting self-reported motivations).

Understanding how different components of nature give rise to psychological benefits is a key question in environmental psychology. The majority of respondents agreed positively with the statements that: watching birds in their garden made them feel relaxed and connected to nature. These feelings increased in people who noticed birds around them for a greater proportion of the day and who fed birds regularly. Stress is a major contributor towards mental health issues such as depression and anxiety [50]. Here we show that the act of maintaining and watching a bird feeder increased self-reported feeling of relaxation, so contributing towards reduced levels of stress. Although we do not show causation, we do not believe that it is too great a leap to conclude that people who feed birds more regularly and feel connected to nature from doing so, feel a deeper connection to nature. Watching birds at feeders and listening to their song provide opportunities to reinforce this connection within one’s own garden [51–52]. Estimates have been made of how much people pay to receive these and other benefits: for example, £240–290 million is spent annually on bird seed in the UK, whilst the bird food industry in the US is estimated to be worth $4.5 billion [23]. As future research explores and quantifies the mental health benefits of engaging with different aspects of nature, these values may be seen as cost efficient investments.

We found that the perception that there is insufficient food available in the natural environment increased with the frequency of bird feeding. While there are doubtless complex relationships between people’s perceptions and actions, this would suggest that these participants believe that birds benefit from supplementary food. Although the casual relationships are not easy to disentangle, at face value this would imply that a concern about bird welfare is a strong motivation behind bird feeding. Indeed, many people feel passionately about the welfare of their garden birds [27], shown here by their willingness also to invest time in offsetting associated risks, such as by following best practise guides (e.g. [37]) to reduce the risk of the spread of disease. Encouragingly we found that 58% of people agreed with the statement that they regularly wash their feeders. However, this figure decreased in younger people and those that only fed birds irregularly, suggesting that people’s willingness to invest in improving avian hygiene may be related to their availability of leisure time.

There is increasing evidence that the greater a person’s orientation towards nature the more they are motivated towards experiencing it, and that this can be a stronger motivation than their opportunity for doing so [38]. Although we did not measure orientation and opportunity directly, we show that people who fed birds regularly would be willing to do so even if there were none currently in the garden, and were less willing to lose the closer and more reliable human-wildlife interaction a bird feeder provides such as by planting bird friendly plants. These feelings decreased with levels of bird feeding, suggesting that people who fed birds regularly were more orientated towards seeking this nature interaction even when there was less immediate opportunity for doing so. Although we show that bird feeding is an expression of nature orientation it is important to acknowledge that it is only one of many different forms of connection to nature.

If feeding birds provides psychological benefits to so many people, then an obvious question remains: why don’t more people do it? Of the people in this study that never provided food only 22% stated that they were not interested in so doing. The strongest indicator that we found of a failure to feed birds was simply that people didn’t remember to do so, especially in respondents under 40 years who are likely to spend less of their leisure time around the home than older respondents [53]. In line with other studies we found that the regularity of bird feeding increased with age (e.g. [20–21]), with respondent’s over 40 years feeling more relaxed when watching birds in their garden than younger counterparts. Older participants’ preference for low arousal (e.g. relaxation) over high arousal (e.g. excitement) emotions may increase in later life [54], suggesting that the benefits of watching birds, and people’s relationship to nature in general, may vary across a person’s life [55]. We did not find gender or income to be significant predictors of motivation in any model, suggesting that amongst people who feed birds such disparities are not important drivers.

In a world where people live increasingly urbanized lifestyles, the nature around where they live and work forms a critical component of their daily nature interaction. A major challenge in harnessing people’s interest in local and broader conservation issues is that many people simply do not notice the nature that is around them [40]. A bird feeder has the potential to be a powerful tool for people to make this connection, because it provides a focal location where people both expect to and are able to observe birds and their behaviours. However, the avian community level impacts of bird feeding vary geographically [56–57] and as a consequence the activity is either supported or discouraged by relevant national conservation organisations (reviewed [27]). Whatever the position, the large number of people engaged in providing food for wild birds suggests that there is a general desire within the wider population to engage with the wildlife around them. Understanding people’s motivations behind bird feeding can open the door to public conversations about conservation management strategies at the local, national and interational levels. Further, if conservation organisations and city planners can maximise the benefits that engaging with wildlife brings then the nature where people live has the potential to contribute towards increased personal and social well-being.

## Supporting Information

S1 File Test of whether two methods of data collection were comparable **(Appendix A).** Birds and you **(Table A)**. Birds in your garden **(Table B).** Why you don’t feed birds **(Table C)**. Birds at your feeder **(Table D).** About you **(Table E)**. Demographic breakdown of the respondents, with comparative nationwide data from UK Census 2011 **(Table Fa),** nature awareness of respondents **(Table Fb).**
(DOCX)
