# Supplementary material for: Urban Bird Feeding: Connecting People with Nature
Source: PLoS One. 2016 Jul 18;11(7):e0158717. doi: 10.1371/journal.pone.0158717 (PMC4948881; doi:10.1371/journal.pone.0158717)
Supplement: S1 File — Test of whether two methods of data collection were comparable (Appendix A). Birds and you (Table A). Birds in your garden (Table B). Why you don’t feed birds (Table C). Birds at your feeder (Table D). About you (Table E). Demographic breakdown of the respondents, with comparative nationwide data from UK Census 2011 (Table Fa), nature awareness of respondents (Table Fb). (DOCX) [file pone.0158717.s001.docx]

**Supplementary Information**

**APPENDIX A**

We tested whether our two methods of data collection were comparable. We did this by pooling the answers from all nine Likert statements, before building an ordinal mixed effect model to test whether there was a difference in responses to statements between our two methods of data collection (two-level factor). The statement id was included as a random factor. We did not find any difference in responses across our two methods of data collection (coefficient = 0.02 ± 0.04 (SE), p = 0.7).

**Birds and people survey**

The survey consisted of seven sections; statements from the analysis appeared in five of the sections and are reported here in as they appeared in the survey. Two other sections are published separately in: Cox & Gaston 2015. Likeability of garden birds: importance of species knowledge and richness in connecting people to nature. Plos One.

**Table A. Q1. Birds and you**: Establish baseline information about household bird related activities**.** Close-ended questions collecting data on the demographics behind who feeds the birds.

| **Question** | **Response options** |
| --- | --- |
| *Birds and you* |  |
| 1. How often do you *usually* put out food for birds? (Please tick one) | Daily, Weekly, Monthly, Less than once a month, Never |
| 1. In which season(s) do you *usually* put out food for birds? (Please tick as appropriate) | Winter, Spring, Summer, Autumn |
| 1. When do you notice birds where you live and/or work? (Please tick as appropriate) | Morning, Lunchtime, Afternoon, Evening, I don’t really notice birds |
| *About you* |  |
| 1. What is your age range? | Five year bands: >20-25 years; 25-30 years, etc. Until 70+ years. |
| 1. What is your gender? | Female / male |
| 1. What is your postcode? |  |

From each of the following three sections respondents were asked to score Likert statements on a five-point scale from strongly dislike to strongly like (n = 331). Three statements each related to three possible motivations behind bird feeding: psychological benefits; welfare issues and nature orientation. We also included two statements to understand why people don’t feed birds, and one statement to test for cultural independence in our sample. *Statements related directly to bird feeding activities and so were not completed by people who did not feed birds (n = 282).

**Table B. Q2. Birds in your garden.**

Thinking about when you see birds in your garden, please rate the extent to which you agree with each statement.

Note, from a total of eight statements in this section.

| **Statement** |
| --- |
| 1. I feel connected to nature when I watch birds in my garden |
| 1. I feel relaxed when I watch birds in my garden |
| 1. When I can recognise a particular individual bird I feel more connected to it |

If you DO NOT feed birds in your garden please continue to Q3

If you DO feed birds in your garden please go to Q4.

**Table C. Q3. Why you don’t feed birds.**

Thinking about why you don’t put out food for birds, please rate the extent to which you agree with each statement.

Note, from a total of nine statements.

| **Statement** |
| --- |
| 1. There is sufficient food available in the environment that birds don’t need help |
| 1. There are enough people in my neighbourhood who feed birds so I don’t need to |
| 1. I am not interested in feeding birds |

Please go to Q6 (not shown here).

**Table D. Q4. Birds at your feeder**

Thinking about when you put out food for birds, please rate the extent to which you agree with each statement.

Note, from a total of 23 statements.

| **Statement** |
| --- |
| 1. There is sufficient food available in the environment that birds don’t need help |
| 1. To help stop diseases spreading I regularly wash my feeders* |
| 1. Even if there are not many birds I still put out food in my garden* |
| 1. I don’t put out food when there are not many birds in my garden* |
| 1. I do not always remember to put out food* |
| 1. There are enough people in my neighbourhood who feed birds so I don’t need to |
| 1. If I could attract the same number of birds to my garden with bird friendly plants, I would stop putting out food* |
| 1. I feed birds because my neighbours do* |

**Table E. Q7. About you**

| **Question** | **Possible responses** |
| --- | --- |
| What is your age range? | Tick boxes provided for 11 categories: 18-20 years, then fives years up to 70, finally 70+ years. |
| What is your gender? | Female or Male. |
| Including you, how many people live at your home who are (please write in number): | Under 16 years  Over 16 years |
| What is your postcode? |  |

**Table F. a) Demographic breakdown of the respondents, with comparative nationwide data from UK Census 2011. b) Nature awareness of respondents.** We also show the percentage of respondents that answered each factor level. The total number of survey respondents *n* = 331.

a)

| **Gender** | | **Age (years)** | | **Income** |
| --- | --- | --- | --- | --- |
| *Survey* | *National* | *Survey* | *National* | *Per week* |
| Male: 40.0 | 49.1 | 20 to 40: 14.6 | 35.2 | *<£590: 52.0* |
| Female: 60.0 | 50.9 | 40 to 60: 38.8 | 35.3 | £591-£670: 36.0 |
|  |  | >60: 45.6 | 29.5 | £671-£790: 11.5 |
|  |  |  |  | >£791: 0.5 |

b)

| **Feeding** | **Notice** | **Connect** |
| --- | --- | --- |
| Don’t: 12.0 | 0: 4.2 | 1: 3.3 |
| Irregularly: 24.4 | 1: 16.7 | 2: 10.7 |
| Regularly: 60.7 | 2: 32.7 | 3: 10.7 |
|  | 3: 16.1 | 4: 30.2 |
|  | 4: 27.4 | 5: 47.9 |
